# Increasing The Number of Embryos Transferred from Two to Three, Does not Increase Pregnancy Rates in Good Prognosis Patients 

**DOI:** 10.22074/ijfs.2015.4543

**Published:** 2015-10-31

**Authors:** Mahnaz Ashrafi, Tahereh Madani, Mina Movahedi, Arezoo Arabipoor, Leili Karimian, Elaheh Mirzaagha, Mohammad Chehrazi

**Affiliations:** 1 Department of Endocrinology and Female Infertility, Reproductive Biomedicine Research Center, Royan Institute for Reproductive Biomedicine, ACECR, Tehran, Iran; 2 Department of Obstetrics and Gynecology, Faculty of Medicine, Iran University of Medical Science, Tehran, Iran; 3 Department of Embryology, Reproductive Biomedicine Research Center, Royan Institute for Reproductive Biomedicine, ACECR, Tehran, Iran; 4 Department of Epidemiology and Reproductive Health, Reproductive Epidemiology Research Center, Royan Institute for Reproductive Biomedicine, ACECR, Tehran, Iran

**Keywords:** Embryo Transfer, Sperm Injections, Intracytoplasmic, Live Birth Rate

## Abstract

**Background:**

To compare the pregnancy outcomes after two embryos versus three embryos transfers (ETs) in women undergoing *in vitro* fertilization (IVF)/intracytoplasmic sperm injection (ICSI) cycles.

**Materials and Methods:**

This retrospective study was performed on three hundred eighty seven women with primary infertility and with at least one fresh embryo in good quality in order to transfer at each IVF/ICSI cycle, from September 2006 to June 2010. Patients were categorized into two groups according to the number of ET as follows: ET2 and ET3 groups, indicating two and three embryos were respectively transferred. Pregnancy outcomes were compared between ET2 and ET3 groups. Chi square and student t tests were used for data analysis.

**Results:**

Clinical pregnancy and live birth rates were similar between two groups. The rates of multiple pregnancies were 27 and 45.2% in ET2 and ET3 groups, respectively. The rate of multiple pregnancies in young women was significantly increased when triple instead of double embryos were transferred. Logistic regression analysis indicated two significant prognostic variables for live birth that included number and quality of transferred embryos; it means that the chance of live birth following ICSI treatment increased 3.2-fold when the embryo with top quality (grade A) was transferred, but the number of ET had an inverse relationship with live birth rate; it means that probability of live birth in women with transfer of two embryos was three times greater than those who had three ET.

**Conclusion:**

Due to the difficulty of implementation of the elective single-ET technique in some infertility centers in the world, we suggest transfer of double instead of triple embryos when at least one good quality embryo is available for transfer in women aged 39 years or younger. However, to reduce the rate of multiple pregnancies, it is recommended to consider the elective single ET strategy.

## Introduction

An important issue in assisted reproductive techniques (ART) is how many embryos could be transferred for each couple. A number of variables considered for a high success rate in *in vitro* fertilization (IVF) treatment may be followed by a high rate of multiple pregnancies. Over 30% of IVF pregnancies are multiples which are associated with increasing maternal and infant morbidity and mortality such as (1, 2) hypertension, polyhydramnios, premature labor, (1) low birth weight, higher perinatal mortality and congenital anomalies (1). Therefore, it is crucial to find proper methods to reduce multiple pregnancies without reducing the overall pregnancy rate. Despite recent recommendations for achieving acceptable pregnancy rate with few multiple pregnancies, one or two good quality embryos needs to be considered for transfer (3-6), but still in some countries including Iran, patients have an impression that increasing the number of embryos transferred is associated with increased pregnancy rate.

Some studies have reported elective singleembryo transfer (eSET) in IVF-intracytoplasmic sperm injection (ICSI) cycles prevents multiple pregnancies without reduction of overall pregnancy rate (3, 4, 6), while some other studies believe that eSET could be associated with a lower pregnancy rate per cycle, especially in an unselected population (5, 7, 8). Despite the efforts have been made in order to limit the incidence of multiple pregnancies after ART (e.g. by SET), the average IVF treatment includes transfer of two, three or sometimes even more embryos into the uterus, while use of eSET in clinical practice has not yet been performed. This may be due to the factors influencing the number of embryos transferred such as cost-effectiveness of eSET technique, professional attitudes and the financial situation of couples. In a recent systematic review, Pandian et al. (8) reported that insufficient data are available on the outcome of two versus three and four ETs policies. In Iran and some other parts of the world where no legal restrictions exist, this is the responsibility of infertility specialists and patients to make decision about the number of embryos transferred with respect to the risks associated with multiple gestations and acceptable pregnancy rates. A recent guideline stating the suitable number of embryos to transfer following an IVF cycle suggests that a maximum of three or four embryos can be transferred in women over the age of 39 (9).

The primary purpose of the present study was to investigate whether increasing the number of embryos transferred from two to three leads to an increase in the overall pregnancy and live birth rates in women undergoing ICSI cycles, then the secondary objective was to evaluate the impact of maternal age on the outcome of IVF/ICSI according to the number of embryos transferred.

## Materials and Methods

This retrospective study was performed at Reproductive Biomedicine Research Center, Royan Institute, Tehran, Iran, from September 2006 to June 2010. The Institutional Review Board of Royan Institute was approved the study. All patients signed a consent form in their initial visit giving permission to use their results without using their names in the future studies. The study population consisted of 387 women with primary infertility and with at least one fresh ET in good quality. Exclusion criteria were as following: use of clomiphene citrate; use of human menopausal gonadotropin (hMG) only in antagonist protocols; women with advanced age (≥40), women with body mass index (BMI) ≥30, as well as history of ovarian hyperstimulation syndrome, uterine factor infertility, severe endometriosis, hydrosalpinges and repeated implantation failure. All patients according to number of ETs were categorized into two groups: ET2 and ET3 groups, indicating two and three embryos were transferred.

In this study, all of the patients underwent a standard long protocol using 500 μg gonadotropinreleasing hormone (GnRH)-a (Buserelin, Superfact, Aventis Pharma Deutshlan, Frankfurt, Germany). Once down-regulation was confirmed by linear endometrium thickness in ultrasonography and serum estradiol concentration <50 pg/ml, the Superfact dose was reduced by one-half (250 μg), and gonadotrophin stimulation with recombinant follicular stimulating hormone (rFSH, Gonal-F, Serono Laboratories Ltd., Geneva, Switzerland) was applied and continued until the day of human chorionic gonadotropin (hCG, IBSA, Switzerland) administration. The first ultrasound scan was performed on day 6 and the dose of rFSH was adjusted according to the ovarian response. When at least two follicles >18 mm were seen, 10000 IU urinary hCG (uhCG, Choriomon, IBSA, Switzerland) was injected intramuscularly and oocyte retrieval was performed 34-36 hours later. The presence of two pronuclei and two polar bodies was assessed 16-18 hours after ICSI. Approximately, 48 hours after injection, embryos were classified based on morphological criteria (10). Embryos with the best morphology and with the most advanced stage of development were selected for transfer. In our institute, the grading of embryos was performed by two embryologists with same background. Two expert clinicians performed the ETs and the difficult ETs were excluded from study.

In our institute, a number of factors, including the patient’s age, cause and duration of infertility, the number and grade of the available embryos and requests of the couples, were taken into consideration in order to decide how many embryos to transfer. ET performed on day 2 or 3 after oocyte retrieval and two or three embryos per patient were transferred. Luteal-phase support was provided with 400 mg vaginal progesterone (Aburaihan co., Tehran, Iran) twice a day until the day of beta-hCG (β-hCG) assay. If the result of β-hCG assay was positive, the same dosage of progesterone was continued up to 10 weeks of gestation. Clinical pregnancy was defined as a positive pregnancy test followed by the presence of fetal sac on transvaginal ultrasound 4 weeks later.

Data were analyzed using the Statistical Package for the Social Sciences 16.0.0 (SPSS, SPSS Inc. Chicago, IL, USA). Demographic factors, reproductive history, ART cycle-specific parameters, and pregnancy outcomes were compared between two groups using the chi-square test for categorical variables and Student t test for continuous variables when data was normally distributed, whereas Mann-Whitney test was used for abnormal cases. All tests were done two tailed. Descriptive statistics are presented as mean ± standard deviation (SD) and percentage. Multiple logistic regression analysis was used to evaluate the association between the number of ET and live birth rate, adjusting for potential confounding variable (age). We used Receiver Operating Curve (ROC) analysis to find the best cut point of age for prediction of live birth by regression equation. A value of P<0.05 was considered to be statistically significant.

## Results

A total of 387 patients were included in this study, among whom 193 patients with two ETs and 194 patients with three ETs. Pregnancy outcomes were compared between ET2 and ET3 groups.

The demographic characteristics are demonstrated in table 1. Two groups had no difference in terms of infertility diagnosis, women’s BMI, infertility duration and the number of previous ART cycles. The mean of maternal age in ET2 group was significantly higher than ET3 group (P<0.001).

**Table 1:** Demographic characteristics of women according to the number of embryos transferred (ET)


Variables	Two ET (n=193)	Three ET (n=194)	P value

Age mean (SD)	29.6 (5.3)	27.5 (3.5)	<0.001
BMI mean (SD)	24.6 (3.4)	24.7 (3.1)	0.48
Infertility duration n (%)	7.0 (5.0)	6.6 (3.8)	0.4
Infertility reason n (%)	-	-	0.59
Male factor	105 (45.4)	113 (58.2)	-
Tubal factor	9 (4.7)	21 (10.8)	-
Ovulatory factor	25 (13)	19 (9.8)	-
Unexplained	20 (10.4)	15 (7.8)	-
Multiple factors	34 (17.6)	26 (13.4)	-
No. of previous cycles n (%)	0.8 (0.5)	0.7 (0.7)	0.2


SD; Standard deviation and BMI; Body mass index.

The mean total dose of rFSH, duration of gonadotropin administration, and the number of MII oocyes were similar between two groups (P=0.7, P=0.6 and P=0.3, respectively). Furthermore, the mean number of oocytes retrieved and total embryos were significantly higher in ET3 group compared to the ET2 group (P=0.001, Table 2). Chisquare test showed that the percent of patients with Grade C ET in ET3 group was higher significantly (P=0.02).

Our results showed that pregnancy rates in patients with two and three ETs were similar (P=0.7). There was also no significant differences in terms of live birth (P=0.4), miscarriage and intrauterine death rate between two groups. Multiple pregnancy rate was significantly higher in the ET3 group compared to ET2 group (Table 3).

The cut point for maternal age obtained by ROC analysis for clinical pregnancy rate was 33 years. Logistic regression analysis revealed that only age was predictable for clinical pregnancy rate in stimulating ICSI cycles [Odds ratio (OR): 1.6, Confidence interval (CI):1.05-2.8] (Table 4). Furthermore, logistic regression analysis for predictive factors of live birth rate showed that the quality of transferred embryos and number of ET were significantly predictable, so that the quality of ET was directly related to the live birth, but the number of ET had an inverse relationship with this variable (Table 5).

As shown in table 6, live birth rates were similar between ET2 and ET3 in women younger and older than 33 years old. Therefore, a reduction in number of embryos transferred did not decrease the clinical pregnancy and live birth rates in both age levels.

**Table 2:** Characteristics of ICSI cycles of study population according to the number of embryos transferred (ET)


Variables	Two ET (n=193)	Three ET (n=194)	Overall P value

No. of total gonadotropins (75 IU/Amp)	26.0 (12.7)	25.7 (9.2)	0.7
Duration of stimulation (days)	10.2 (2.0)	10.3 (2.1)	0.6
No. of oocytes retrieved	9.5 (6.1)	11.8 (5.8)	<0.001
No. of M2 oocytes	7.5 (5.4)	8.0 (4.5)	0.3
No. of embryos	4.8 (3.9)	6.2 (3.2)	<0.001
Quality of transferred embryos	-	-	0.02
Two or three ET (grade A) n (%)	81 (42)	79 (40.7)	-
Two or three ET (grade B) n (%)	110 (57)	100 (51.5)	-
One ET (grade A or B) and one or two ET grade C	2 (1)	15 (7.8)	-
Day of embryos transferred	2.2 (0.4)	2.3 (0.5)	0.01


Data are presented as mean ± SD. ICSI; Intracytoplsamic sperm injection, SD; Standard deviation and M2; Metaphase II.

**Table 3:** ICSI outcomes according to the number of embryos transferred (ET)


Variables	Two ET (n=193)	Three ET (n=194)	Overall P value

Fertilization rate mean (SD)	61.5 (30.5)	61.2 (35.0)	0.9
Implantation rate mean (SD)	26.1 (35.7)	20.0 (29.5)	0.07
Clinical pregnancy rate n (%)	78 (40.4)	73 (37.6)	0.7
Multiple pregnancy rate n (%)	21 (27)	33 (45.2)	0.01
Miscarriage rate n (%)	6 (3.1)	10 (5.1)	0.3
Live birth rate n (%)	69 (35.7)	60 (30.9)	0.4
Intrauterine fetal death n (%)	3 (1.5)	3 (1.5)	NS*


*NS; Not significant, ICSI; Intracytoplsamic sperm injection and SD; Standard deviation.

**Table 4:** Logistic regression analysis for predicting the clinical pregnancy rate in ICSI cycles


Variable	OR	95% CI	P value

**Age**			
**<33**	1.6	(1.05-2.8)	0.05
**≥ 33 years old**	Reference group	-	-


ICSI; Intracytoplsamic sperm injection, OR; Odds ratio and CI; Confidence interval.

**Table 5:** Logistic regression analysis for predicting the live birth rate in ICSI cycles


Variable	OR	95% CI	P value

Quality of transferred embryos			
Grade A	3.1	(1.1-9.0)	0.02
Grade B	2.2	(1.07-7.0)	0.05
Grade C	Reference group	-	-
Number of embryos transferred			
Two embryos	3.1	(1.09-9.2)	0.03
Three embryos	Reference group	-	-


ICSI; Intracytoplsamic sperm injection, OR; Odds ratio and CI; Confidence interval.

**Table 6:** Age related results according to number of embryos transferred (ET)


<33 years: No. of cases	Two ET n=136	Three ET n=132	P value

Implantation rate mean (SD)	29.0 (36.2)	20.8 (30.2)	NS*
Pregnancy rate n (%)	61 (44.8)	57 (43.1)	NS
Live birth rate n (%)	53 (38.9)	46 (34.8)	NS
Multiple pregnancy rate (%)	17 (27.8)	30 (52.6)	0.001
**≥33 years: No. of cases**	** n=57 **	**n=62**	
Implantation rate mean (SD)	19.2 (33.7)	11.7 (20.2)	NS
Pregnancy rate n (%)	17 (29.8)	17 (28)	NS
Live birth rate n (%)	16 (28.0)	14 (22.5)	NS
Multiple pregnancy rate n (%)	4 (23.5)	3 (17.6)	NS


*NS; Not significant and SD; Standard deviation.

## Discussion

Our study indicated that in good prognosis patients aged 39 years or younger, two and three ETs have same pregnancy and live birth rates, while the multiple pregnancy rate was significantly higher in ET3 group; therefore, it is recommended to transfer two instead of three embryos.

Based on a recent guideline, individual IVF-ET centers should evaluate their own data to identify patient-specific, embryo-specific, and cycle-specific to determine factors of implantation and live birth in order to develop ET protocols minimizing the occurrence of multi-fetal gestation, while preserving acceptable overall pregnancy and live birth rates (11).

In a recent study, Min et al. (9) presented a guideline for the number of embryos transferred considering the maternal age; however, this numbers can be different in various infertility centers according to the laws of those countries. In a number of countries, including Norway, Sweden, Denmark, Belgium, England, Italy, Germany, and Australia, the complications associated with multiple pregnancies are reduced through use of SET by legal restrictions, while many other European countries have bordered to a maximum of two ETs.

In other parts of the world like Iran, there is no legal restriction in this regard, and it is the responsibility of infertility specialists and patients to make decision about the number of embryos transferred. Various strategies for eSET depend on different funding methods of infertility treatments. There are countries where the public sector covers the majority of costs, whereas in some other courtiers, patients have to undertake the costs directly or indirectly through private insurance systems. Despite the recent emphasis and supports on eSET (3-6, 12, 13), this technique in not generally used in Iran because of heavy treatment costs. SET in our institute is necessarily for some patients with special conditions such as poor ovarian response and male factor cases.

In agreement with the previous studies (14- 17), we found similar clinical pregnancy and live birth rates in patients with two versus three ETs, but the multiple pregnancy rate in our study was significantly greater in group with three embryos than two embryos transferred. Despite the fact that women in ET3 group were younger than ET2 group, but due to higher number of couples with male infertility in the ET3 group, the number of patients with grade C embryos transferred were significantly higher than ET2 group. Therefore, no significant difference was observed between the two groups in terms of implantation, clinical pregnancy and live birth rates.

We evaluated the influence of maternal age on IVF/ICSI outcome for the number of ET and the obtained data indicated lower rate of multiple pregnancies in group with two embryos transferred as compared to ET3 group with individuals of different ages (younger or older than 33 years), while the pregnancy and live birth rates were similar. In contrast to our results, Giannini et al. (18) showed that in older women (≥35 years), a reduction in the number of embryos transferred significantly decreased the chances of pregnancy.

Logistic regression analysis showed that one significant prognostic variable for clinical pregnancy: maternal age (as categorical variable); it means that in women younger than 33 years old, the chance of clinical pregnancy increased 1.6- fold. Our results were in line with Chuang et al. (19) that age is a good predictor for pregnancy potential. Implantation, pregnancy and live birth rates in women younger than 33 years old in both groups (ET2 and ET3) were higher than older women.

On the other hand, logistic regression analysis indicated two significant prognostic variables for live birth that were quality and number of transferred embryos; it means that the chance of live birth following ICSI treatment increased 3.2-fold when the embryo with top quality (grade A) was transferred. Our result is in agreement to Dennis et al. (20) study, in which they suggested that embryo grade is a significant predictor for live birth rate. But the number of ET had an inverse relationship; it means that probability of live birth in women with transfer of two embryos was three times greater than those who had three embryos transferred.

One of the limitations of this study comes from its retrospective nature; however, we tried with regression test to control confounding factors.

Present study was performed in fresh ET cycles, and because the probability of no synchronization between the time of fresh ET and the window of implantation in the endometrium is existed (21), we suggest future study to evaluate the association between number of embryos transferred and live birth rate in cycles with frozen and/or blastocyst stage ETs.

According to a recent guideline, women aged 35 years or younger, in first or second IVF attempt, with at least 2 good quality embryos transferred should be considered as good-prognosis patients, and the eSET strategy should be used in order to avoid multiple pregnancies (9). In our study, the rate of twin pregnancy in women with double ET was 27%, which is not acceptable; therefore, it is recommended that the eSET strategy be considered, although there are no executive arrangements to enable our institution to enforce the eSET strategy. Moreover it is important to remind the patients about the high risk nature of multiple pregnancies that requires equipped labor which may not be available in some rural or smaller areas. Concerns in relation to multiple pregnancies and even twin pregnancy resulting from ART specify the importance of counseling before ET for IVF patients (22).

## Conclusion

Due to the difficulty of implementing the eSET strategy in some infertility centers in the world including Iran, we suggest transferring of a maximum of two embryos with at least one good quality embryo for good prognosis women aged 39 years or younger. However, further clinical trials are required to evaluate the effect of the number of embryos transferred (single versus double ET) for women in different age groups using fresh or frozen ET technique.
